# Sanguinarine Inhibits Vascular Endothelial Growth Factor Release by Generation of Reactive Oxygen Species in MCF-7 Human Mammary Adenocarcinoma Cells

**DOI:** 10.1155/2013/517698

**Published:** 2013-05-21

**Authors:** Xian-zhe Dong, Miao Zhang, Kun Wang, Ping Liu, Dai-hong Guo, Xiao-li Zheng, Xiao-yue Ge

**Affiliations:** ^1^Department of Clinical Pharmacology, Chinese PLA General Hospital, Beijing 100853, China; ^2^Chinese PLA General Hospital, Beijing 100853, China; ^3^College of Pharmacy, Tianjin University of Traditional Chinese Medicine, Tianjin 300193, China; ^4^College of Pharmacy, Bengbu Medical College, Bengbu 233030, China

## Abstract

The inhibitory action and the possible mechanism of anticancer compound Sanguinarine (SAN) on vascular endothelial growth factor (VEGF) in human mammary adenocarcinoma cells MCF-7 were evaluated in this study. We exposed MCF-7 to SAN for 24 h, then cell viability was assessed by using the 3-(4,5-dimethylthiazol-2-yl)-2,5-diphenyltetrazolium bromide (MTT) reduction assay. Human VEGF was measured using a paired antibody quantitative ELISA kit, relative expression of VEGF mRNA was calculated using the real-time PCR studies, and the effect of SAN on the reactive oxygen species (ROS) level was detected by the flow cytometer. Treatment with SAN remarkably inhibited growth of MCF-7 cells and induced cell apoptosis. We found that VEGF release was stimulated by subtoxic concentrations of SAN and inhibited by high dose of SAN, SAN-evoked VEGF release was mimicked by low concentration of H_2_O_2_, and SAN-regulated VEGF inhibition was accompanied by increasing of ROS; these changes were abolished by antioxidant. High concentration of SAN inhibited VEGF mRNA expression in MCF-7 cultures, suggesting an effect at transcriptional level, and was also abolished by antioxidant. The present findings indicated that the regulation of VEGF expression and release from MCF-7 cells were possibly through reactive oxygen species evoked by SAN.

## 1. Introduction

**Figure 1:**
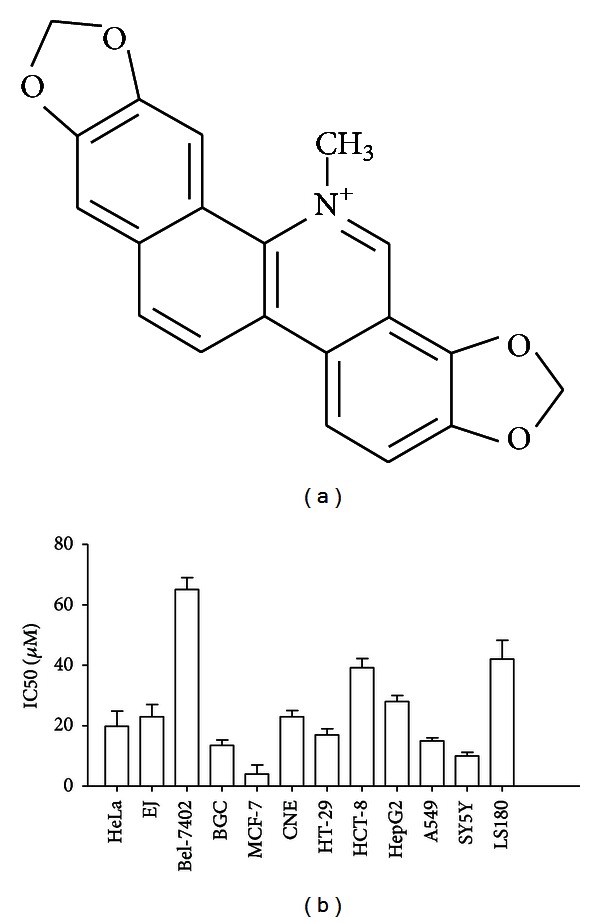
Growth-inhibitory effect of SAN on the viability of tumor cells evaluated by MTT assay. (a) Chemical structure of SAN. (b) Effect of SAN on proliferation of tumor cells. Each data represented the mean  ±  SD from three independent experiments, each at least in triplicate.

*Sanguinaria canadensis* is a traditional herbal remedy which was produced in the eastern and southern provinces and the long-river basin of China used to treat fever and inflammation. SAN is an alkaloid obtained from the bloodroot plant *Sanguinaria canadensis* with the chemical constitution as Figure 1(a) and has beneficial effects on inflammatory disorders. Previous reports have demonstrated that SAN also exhibits anticancer properties. Extensive work over the past decade has shown that SAN can induce obvious cytotoxicity in many tumor cell lines, such as human colon cancer cells, oral squamous cell carcinoma cell line, prostate cancer cell, human osteosarcoma cells, and human gastric adenocarcinoma [1–6].

In our previous experiments, we determined the antitumors effect of SAN on a variety of tumor cells using the 3-(4,5-dimethylthiazol-2-yl)-2,5-diphenyltetra-zolium bromide (MTT) assay to investigate which cancer cell line was the most sensitive to SAN at the same condition. The cell lines were included Bel-7402 (human liver cancer), A549 (human lung cancer), HCT-8 and HT-29 (human colon carcinoma), BGC (human gastric cancer), LS180 (human colorectal), HeLa (human cervical), HepG2 (human hepatocellular carcinoma), EJ (human bladder cancer), SY5Y (human neuroblastoma), CNE (human nasopharyngeal carcinoma), and MCF-7 (human breast cancer). The results showed that MCF-7 cell line was the most sensitive cell line to the antitumor effect of SAN, the IC50 (50% inhibition concentration) of SAN was only 4 *μ*M (Figure 1(b)). So the purpose of the present study was to clarify the related mechanism of SAN on human bladder cancer MCF-7 cells.

## 2. Methods

### 2.1. Cell Culture and Treatment

The human mammary adenocarcinoma MCF-7 was obtained from the Department of Obstetrics and Gynecology in Chinese PLA General Hospital and maintained in Dulbecco's Modified Eagle Medium (DMEM) supplemented with 10% FBS (heat inactivated at 56°C for 30 min) and 100 U/mL of penicillin and 100 U/mL streptomycin, in a 37°C incubator with a humidified, 5% CO_2_ atmosphere. Confluent MCF-7 cells were seeded into 96-well plates at a density of 1 × 10^4^ cells/well. After 24 h, the cells were exposed to various concentrations of SAN and incubated for 24 h. SAN was dissolved in dimethyl sulfoxide (DMSO) before added in cell. Final concentrations of DMSO were always less than 0.01%, which was proved to have no effects on cell viability. The absorbance was read at 570 nm with DMSO as the blank.

### 2.2. Cell Viability

The cell viability was determined by MTT assay. Briefly, 20 *μ*L of MTT solution (2 mg/mL in PBS) was added to the culture medium at a final concentration of 0.5 mg·mL^−1^ and incubated at 37°C for 4 h. Then the supernatants were aspirated carefully, 150 *μ*L of DMSO was added to each well to dissolve the reaction product of MTT, and the OD was spectrophotometrically measured at 570 nm, with DMSO as a blank. Viability was expressed as percentage of the values in vehicle-treated (basal) cultures, set to 100%.

### 2.3. Effect of SAN on Growth Curve

Cell growth curves were measured by the MTT assay. Cells were seeded into 96-well plates at a density of 1 × 10^4^ cells/well, treated with or without different concentrations of SAN, and then the cell viability at different time points was determined. The cell growth curves were drawn by the cell survival rates and the time points. The viability of each group at 0 h was supposed as “100%.”

### 2.4. Flow Cytometric Detection of Apoptotic Cells

Cell apoptosis was measured using Annexin-V-FITC/PI apoptosis detection kit. 5 × 10^6^ cells were plated in 6-well plates per well. After treatment with SAN for 24 h, cells were harvested and washed in PBS, and then centrifuged at 1000 ×g for 5 min. The cell pellet was resuspended in the Annexin-V-FITC/PI labeling solution, mixed gently, and incubated for 15 min at room temperature in darkness. Cells were then analyzed in a Becton Dickinson flow cytometer (USA), and each sample collected ten thousand cells.

### 2.5. Intracellular ROS Quantification

The level of intracellular reactive oxygen species (ROS) was determined by the change of fluorescent probe dichlorofluorescein diacetate (DCFH-DA). Briefly, 6 × 10^4^ MCF-7 cells were cultured into 6-well plate and were treated with indicated concentrations of SAN (1, 2, 4, and 8 *μ*M) for 24 h. Cells were trypsinized and washed with PBS, then incubated with 10 mM DCFH-DA for 30 min at 37°C. Subsequently, cells were washed twice with PBS and analyzed by flow cytometer. The protective effect of NAC was determined by adding 10 mM NAC or 20 kU/L SOD with SAN to the MCF-7 cells before the ROS measurement. Ten mM NAC or 20 kU/L SOD could inverse the inhibition effect of SAN on MCF-7 cell growth most significantly in the preliminary experiment. 

### 2.6. Enzyme-Linked Immunosorbent Assay

Culture media were collected following treatments and promptly stored at −80°C until use in the assay [7]. Human VEGF was measured using commercially available ELISA kit purchased by Invitrogen according to the manufacturer's instructions. The assay sensitivities of VEGF were 5 pg·mL^−1^; data were expressed as pg·mL^−1^.

### 2.7. Reverse Transcriptase-Polymerase Chain Reaction (RT-PCR)

Total RNA was extracted from cultured cells with Trizol (Gibco BRL) according to the manufacturer's instructions. cDNA was synthesized from 1 *μ*g of purified RNA with random primer with the use of the First Strand Synthesis Kit (ReverTra Ace-a-, TOYOBO CO, JAPAN). Human *β*-actin and VEGF primer pairs used were synthesized by BM (Biomed, China) and described as follows. *β*-actin: forward 5-GGACATCCGCAAAGACCTGTA-3, reverse 5-ACATCTGCTGGAAGGTGG ACA-3; VEGF: forward 5-TTGCTGCTCTACCTCCAC-3, reverse 5-AAATGCTTTCTCCGCTCT-3. The PCR reaction was performed using the SYBR Green real-time PCR Master Mix (Toyobo, Japan) to detect abundance of PCR products among samples. The cycling conditions were, 50°C for 2 min and 95°C for 3 min, followed by 40 cycles of 95°C for 30 s, 60°C for 1 min, and 72°C for 30 s. VEGF gene normalized against *β*-actin which was chosen as an internal control and carried out from the same sample. Relative expression of VEGF mRNA was calculated using the 2^−Δ(ΔCT)^ comparative method [8]. All quantities were expressed as n-fold relative to the calibrator (control values which was defined as a value of “1.0”).

### 2.8. Statistical Analysis

Values are expressed as mean  ±  S.D. Statistical analysis was done by using the SPSS 16.0 software. One-way analysis of variance (ANOVA) followed by Tukey's post hoc test was used for multigroup comparisons. Values of *P* < 0.05 and *P* < 0.01 were considered statistically significant.

## 3. Result

### 3.1. Effect of SAN on Cell Growth

**Figure 2:**
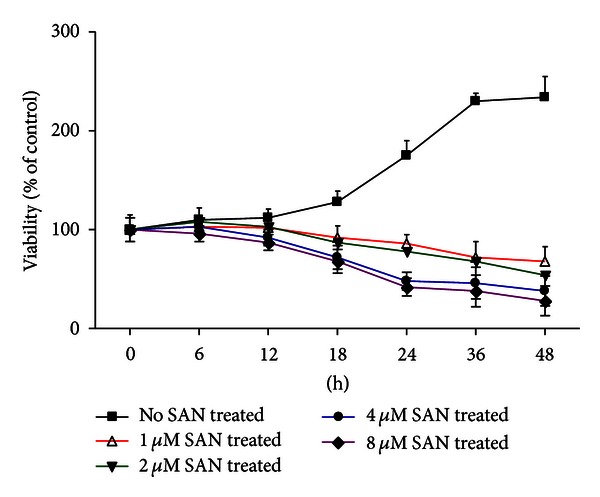
Effect of SAN on cell growth in MCF-7 cells by MTT assay. MCF-7 cells were treated with SAN for 24 h. SAN treatment decreased the growth in MCF-7 cells.

From the growth curve (Figure 2), in the control group, cells were growing in normal form and speed; cellular proliferation was going into logarithmic phase after 18 hours from seeding in plates. After different concentrations of SAN treatment, the cells growth was suppressed significantly by time dependent and concentration dependent manner.

### 3.2. Effect of SAN on Cell Apoptosis

**Figure 3:**
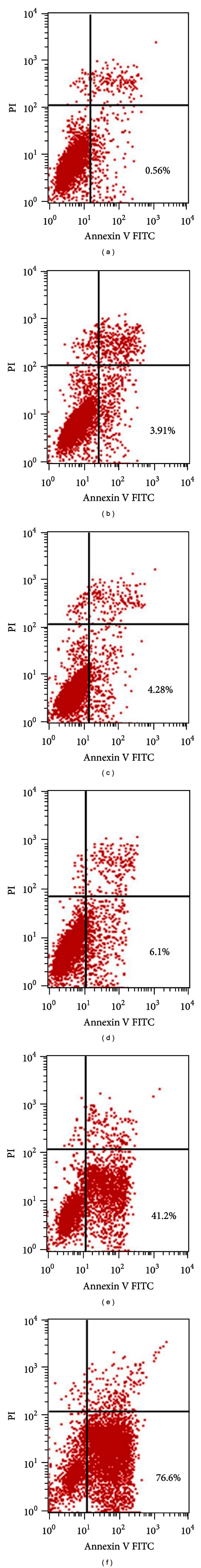
Effect of SAN on apoptosis in MCF-7 cells by flow cytometry. Annexin-V-FITC/PI analysis of MCF-7 cells treated with SAN for 24 h; SAN treatment increased the apoptosis rate in MCF-7 cells. (a): Control; (b): 0.5 *μ*M; (c): 1 *μ*M; (d): 2 *μ*M; (e): 4 *μ*M; (f): 8 *μ*M.

The staining assay was used to evaluate the apoptosis in MCF-7 cells. As shown in Figure 3, control cells without the treatment with SAN exhibited intact cell membrane (A). The cells, treated with SAN (0.5, 1, 2, 4, and 8 *μ*M), increased the percentage of apoptotic cells from 3.9% to 76.6%, compared to control cells.

### 3.3. Effect of SAN on the Level of VEGF in MCF-7 Cells

**Figure 4:**
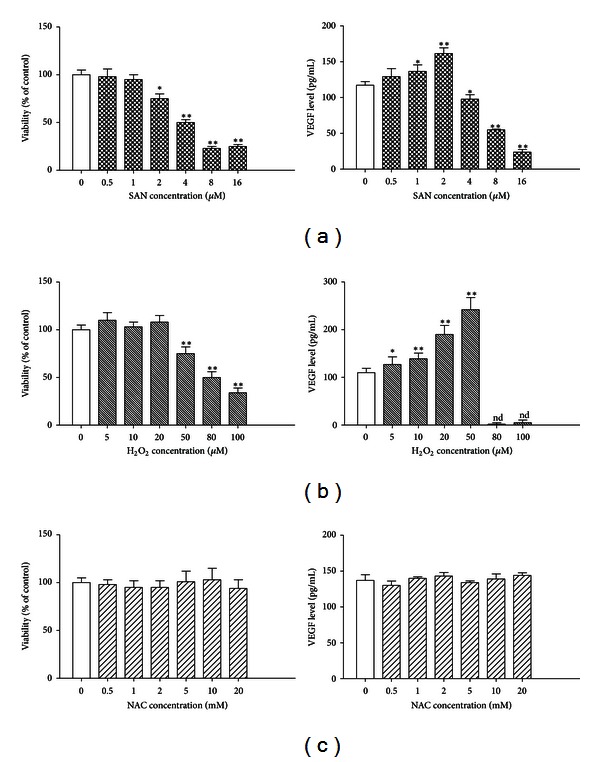
SAN regulates VEGF release from MCF-7 cells. Effects of increasing concentrations of SAN (a), H_2_O_2_ (b), and NAC (c) on cell viability (MTT test) and VEGF release in MCF-7 cultures. Each histogram is the mean  ±  SD of three independent experiments performed in quadruplicate. “nd” was not detectable. Compared with normal group, *P < 0.05, **P < 0.01.

Initially, the cytotoxicity of SAN was investigated with different doses of SAN in human MCF-7 cells by MTT assay. The effects of SAN on the level of the VEGF were also examined in MCF-7 cells. SAN inhibited the viability of MCF-7 cells in a dose-dependent manner. As shown in Figure 4(a), low dose of SAN (0.5 *μ*M) had no effect on the level of VEGF, then 1 and 2 *μ*M SAN treatment increased the VEGF level. On the contrary, it decreased the levels of VEGF after the treatment of high-dose SAN (4, 8, and 16 *μ*M) comparison with the normal control group. H_2_O_2_ mimicked the effects of low dose SAN (0.5–2 *μ*M) on VEGF release. The 24 h exposure to H_2_O_2_ (5–100 *μ*M) stimulated the release of VEGF from MCF-7 cultures in a concentration dependent fashion. Maximal effect was observed at 50 *μ*M. As assessed with the MTT assay, concentrations from 50 *μ*M to 100 *μ*M affected cell viability (Figure 4(b)). Whereas NAC (0.5 mM to 20 mM) did not result in a significant change of cell viability and VEGF level (Figure 4(c)).

### 3.4. SAN Enhances ROS Levels in MCF-7 Cells

**Figure 5:**
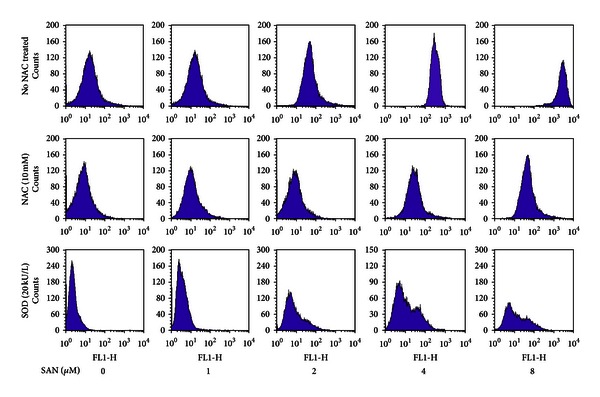
Effect of SAN on ROS levels in MCF-7 cells using a flow cytometry. 10000 MCF-7 cells were cultured into 6-well plates and were treated with indicated concentrations of SAN for 24 h. In the fluorescence intensity of NAC against ROS in MCF-7 cells which treated with SAN, the antioxidant NAC (10 mM) was also added together with the SAN for 24 h.

To investigate whether SAN can increase ROS levels, we examined the intracellular ROS production using a fluorescent probe, DCFH-DA. As shown in Figure 5, after SAN treatment, the fluorescence intensity increased remarkably compared with control group (*P* < 0.01). In addition, the antioxidants, 10 mM of NAC or 20 kU/L of SOD, were added together with the SAN; results showed that compared with cells treated with SAN alone groups, the fluorescence intensity reduced remarkably, which showed that the NAC and SOD can block the effect of SAN in the enhancement of intracellular ROS levels.

### 3.5. ROS Are Responsible for SAN-Induced VEGF Change in MCF-7 Cells

**Figure 6:**
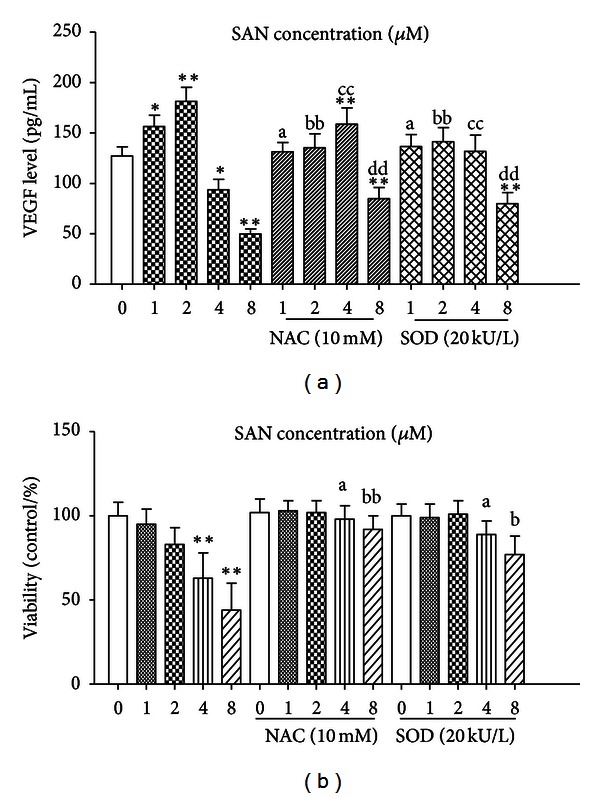
NAC and SOD inverse the effects of SAN on VEGF release from MCF-7 cells. Each histogram is the mean  ±  SD of three independent experiments. (a) Compared with normal group, *P < 0.05, **P < 0.01; compared with SAN treatment alone group, a means *P* < 0.05, bb, cc, dd mean *P* < 0.01. (b) Compared with normal group, **P < 0.01; compared with SAN treatment alone group, a, b mean *P* < 0.05, bb mean *P* < 0.01.

We evaluated the effect of SAN on VEGF release in MCF-7 cells.  Figure 6 showed that SAN either alone or in combination with NAC or SOD had quite different effects in regulating VEGF release in MCF-7 cells. The VEGF release increased significantly when the concentration of SAN was low and inversed by NAC and SOD. But the VEGF release decreased when the concentration of SAN (4 and 8 *μ*M) gradually increased on the contrary. When added to a final concentration of 10 mM NAC or 20 kU SOD, the VEGF release of 8 *μ*M SAN treatment group increased but still lower than normal control, but the VEGF levels of 4 *μ*M SAN treatment group increased unexpectedly even higher than normal control (Figure 6(a)). Simultaneously, NAC and SOD can suppress SAN-induced cell death in MCF-7 cells (Figure 6(b)). The results revealed that NAC and SOD can suppress low concentration of SAN-induced VEGF release and raise high concentration of SAN-induced VEGF inhibition in MCF-7 cells. ROS generation is crucial for SAN-induced VEGF release.

### 3.6. SAN Inhibited Steady-State Levels of VEGF mRNA in MCF-7 Cultures

**Figure 7:**
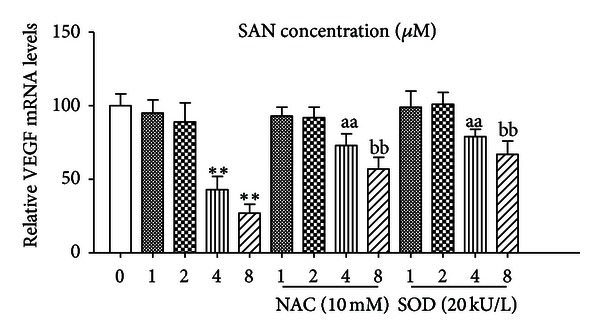
SAN decrease VEGF mRNA expression. MCF-7 cells were treated with SAN (with the indicated concentration) for 24 h. Total mRNA was extracted, and relative VEGF mRNA amounts were measured by real-time PCR. VEGF mRNA levels are expressed as fold change over the basal after normalizing to *β*-actin. VEGF mRNA levels were suppressed by 4 *μ*M and 8 *μ*M of SAN in MCF-7 cultures, and NAC and SOD can block the VEGF mRNA suppression. Each bar is the mean  ±  SD of three independent experiments. Statistical different from basal (vehicle-treated) was calculated by Dunnett's test after ANOVA, **P < 0.01; compared with SAN treatment alone group, aa, bb mean *P* < 0.01.

MCF-7 cell cultures were exposed to SAN (1, 2, 4, 8 *μ*M) for 24 h. Total RNAs were extracted and relative VEGF mRNA amounts were measured by real-time PCR and normalized to *β*-actin mRNA. VEGF mRNA levels were found to be significantly downregulated at 4 *μ*M and 8 *μ*M groups in MCF-7 cultures (over vehicle-treated basal). 10 mM NAC and 20 kU/L SOD can block the VEGF mRNA supression which were induced by 4 *μ*M and 8 *μ*M SAN in MCF-7 cells (*P* < 0.01). But there were no effects of low concentration of SAN (1 *μ*M and 2 *μ*M groups) on the VEGF mRNA expression (Figure 7). May be high density ROS generation is serviceable for SAN-induced VEGF mRNA inhibition, low concentration of ROS is useless for VEGF mRNA inhibition induced by SAN. 

## 4. Discussion

Human mammary adenocarcinoma cells MCF-7 is a classical cell line obtained from breast cancer patient, and it is used in various kinds of studies of breast cancer. MCF-7 has character of differentiated mammary epithelial cells, such as product estradiol, expressing the receptors of estrogen and progestogen [9]. On the other hand, MCF-7 cell line was more sensitive to the antitumor effect of SAN than other tumor cell lines in this experiment, so we selected the MCF-7 cells and expected that SAN could be effective for the treatment of mammary adenocarcinoma someday clinically.

Apoptosis (or programmed cell death) is a physiological mechanism that is crucial for normal development of organisms during embryogenesis, maintenance of tissue homeostasis in adults, and elimination of diseased or otherwise harmful cells during pathogenesis [10]. Dysregulated apoptosis has been implicated in many human diseases, including neurodegenerative diseases such as Alzheimer disease and Huntington disease, ischemic damage, autoimmune disorders, and several forms of cancer [11]. In this study, SAN was shown to enhance apoptosis and ROS content in human mammary adenocarcinoma MCF-7 cells, and VEGF inhibition depending on ROS generation was involved in this effect, and the inhibitor of ROS can protect MCF-7 cells from apoptosis.

ROS are recently proposed to be involved in tumor metastasis which is a complicated process including epithelial-mesenchymal transition (EMT), migration, invasion of the tumor cells, and angiogenesis around the tumor lesion [12]. Our results suggested that the activity of ROS was increased significantly in MCF-7 cells after the treatment of SAN showed that SAN could mediate ROS production. And the apoptosis rate was increased following the increase of content of ROS and could be protected by NAC.

Moreover, our results showed that SAN deactivated the activity of VEGF significantly at the high concentration but raised the release of VEGF at the subcytotoxic concentration. Vascular endothelial growth factor (VEGF), the most active endogenous proangiogenic factor and an endothelial cell-specific mitogen, is involved in angiogenesis in various types of tumors. In cancer cells, both VEGF and VEGF receptors (VEGFR) are overexpressed. VEGF promotes cancer growth, dissemination, and metastasis, and its expression level is positively correlated with the prognosis of cancer in diagnosed patients or animal models [13–15].

But why is the effect of SAN on VEGF release in MCF-7 cells quite different in different concentrations of SAN group, and also in combination with NAC or SOD group? We found that when the content of ROS was inhibited by NAC or SOD, the increased VEGF expression induced by low concentration of SAN (1 and 2 *μ*M) was inhibited immediately. This phenomenon may be due to that the low concentration of SAN induced low content of ROS, the low concentration of ROS promoted the release of VEGF, and antioxidant canceled the generation of ROS, then the intracellular ROS approached normal level, so the expression of VEGF was reduced; in other words, the low concentration of ROS is critical for the release of VEGF. As shown from the results, 2 *μ*M SAN treatment of SAN induced production of VEGF, but not in the mRNA level change. This may be because that low level of ROS could not inhibit the mRNA of VEGF, and fortunately, could activate the upstream signal transduction pathways of VEGF in MCF-7 cell. On the one hand, ROS can also upregulate VEGF secretion and VEGFR expression through induction of transcription factors HIF-1, suppressing HIF degradation increasing HIF production. On the other hand, ROS mediate transcriptional activation via NF-*κ*B and translational activation via PI3K/AKt/4E-BP1 pathway [16].

Actually, it has also been reported that ROS play a critical role in the expression of VEGF. VEGF is supposed to utilize ROS as a messenger intermediates downstream of the VEGF receptor-2. Inhalation of diesel exhaust particles induction of ROS in capillary-like endothelial tubes leads to VEGF-A expression. Atorvastatin reduces VEGF expression in human nonsmall cell lung carcinomas (NSCLCs) via inhibition of ROS production. Psoriasin may therefore play a role in breast cancer progression by promoting oxidative stress response and angiogenesis. Tetramethylpyrazine inhibits hypoxia-induced pulmonary vascular leakage in rats via the ROS-HIF-VEGF pathway [17–22].

But the regulation of SAN on VEGF did not in a concentration dependent manner; SAN deactivated the activity of VEGF at the high concentration (4 and 8 *μ*M), and the inhibition was inversed by 10 mM of NAC and this concentration of NAC, could block the high level of ROS in MCF-7 cells induced by SAN markedly. Combining the experimental results that SAN upregulated the content of intracellular ROS, we supposed that high concentration of ROS was not as the signaling molecules or second messengers but promoted oxidative stress response simultaneously and inhibited the expression of VEGF mRNA. And after the treatment of antioxidants, the levels of intracellular ROS were weakened and approached to low levels, and low levels of ROS did not inhibit VEGF expression but promoted the release of VEGF. Accordingly, we observed the interesting phenomenon.

Therefore, we concluded that SAN induced apoptosis in MCF-7 cells via the inactivation of VEGF through an ROS-dependent manner. Then why does 2 *μ*M of SAN induce protein levels of VEGF but still does not promote cell survival? Is there other mechanisms that participate in the apoptosis of MCF-7 induced by SAN? And how about the borderline between the positive and negative effect on VEGF levels induced by ROS? What is the least concentration of ROS that can suppress the release of VEGF? To resolve the above issues, we may need more studies in the future.
